# Sleep duration on a population of children referred to sleep study - cross-sectional data from 2003 to 2009

**DOI:** 10.5935/1984-0063.20190094

**Published:** 2019

**Authors:** Cristiane Fumo-dos-Santos, Marcia Pradella-Hallinan, Beatriz Neuhaus Barbisan, Sergio Tufik, Gustavo Antonio Moreira

**Affiliations:** 1 Universade Federal de São Paulo, Departamento de Psicobiologia - São Paulo - São Paulo - Brazil.; 2 Universade Federal de São Paulo, Departamento de Pediatria - São Paulo - São Paulo - Brazil.

**Keywords:** Sleep, Child, Habits, School

## Abstract

**Introduction:**

Sleep is essential for human beings, especially children. Insufficient sleep is linked to somatic and psychological problems. This study aims to describe nocturnal sleep patterns in children aged 7 to 13 years and investigate if sex or weekdays influence sleep habits. It also analyses factors associated with sleep length and the difference between sleep habits on weekends and weekdays.

**Methods:**

A retrospective cross-sectional study with questionnaires from children with sleep complaints referred to our service (December 2003 to June 2009) in Sao Paulo City, Brazil. Median of sleep hours, time going to bed, waking up, and the difference in amount of sleep during weekends and weekdays were calculated. A generalized linear model was used to find associations between covariates and a) sleep hours, and b) sleep weekend minus - weekdays.

**Results:**

We analyzed 577 children (median 9.5 y, 61% boys). Median bedtime was 22h. Median wake up time was 7h on weekdays and 9h on weekends. Median sleep duration was 9.5h during weekdays and 10h on weekends. The median difference in the amount of sleep during weekends and weekdays was 0.5h (IQR=1.5). Shorter sleep duration was associated with age and school schedule. Higher difference weekend - weekdays was associated with older children, girls, and school schedule.

**Conclusion:**

Children 7 to 13 years usually sleep more on weekends. Age, morning and full-time classes are associated with shorter sleep duration on weekdays and higher weekend-weekdays; girls sleep more during weekends.

## INTRODUCTION

Sleep is essential for human beings, especially children, since its duration and quality is strongly associated with physical^1^^,^^2^ and psychological health^3^^,^^4^. Insufficient sleep may lead to behavioral problems^5^, obesity, and higher cardiovascular risk^6^. Sleep is also important for brain maturation^7^, learning^8^, and memory^9^.

Sleep duration in children varies with many factors: age, secular trends, daily habits, sex, social cultural factors, and diseases, among others. Children usually sleep less with increasing age^10^^,^^11^. However, a meta-analysis evaluating sleep parameters from childhood to old age found that children and adolescents had different sleep durations depending on age only on school-days. On non-school-days, sleep duration was the same from childhood to the end of adolescence^12^. Daily habits can interfere with sleep duration as well. Shorter sleep duration in children is associated with watching television for 1.5 hours or more per day^13^. Social and cultural factors also play a role in sleep habits: Chinese children went to bed later and woke up earlier than North-American school-aged children^11^. The American Academy of Sleep Medicine has recommendations of sleep hours according to age: for 6 to 12-year-old children they recommend 9-12 hours of sleep^14^.

Regarding diseases, some might enhance the amount of sleep (such as sleep disorders breathing, periodic leg movement, epilepsy, hypersomnia of central origins, hypersomnia due to medications) while some might lead to insomnia (side effects of medications, primary insomnia, restless leg syndrome, pain, asthma and other atopic diseases)^15^.

There are few studies evaluating the amount of time children sleep in Brazil^16^^-^19. In order to explore this, we studied a population referred to polysomnography for a variety of reasons, but only included those with normal results. We considered that sleep time in children varies with age, sex, body mass index, and daily habits. The aims of this study are: 1) in school age-children refered to PSG with suspected sleep disorders, describe nocturnal sleep duration, bedtime, and wake up time based on home-patterns (questionnaires’ answers), 2) investigate the effect of sex and weekdays on sleep habits, 3) and test the association between school schedules and sleep patterns.

## METHODS

### Study design

In a retrospective cross-sectional study, we investigated the sleep patterns of school aged children with sleep complaints referred to a sleep center in Sao Paulo City, Brazil, due to possible sleep-related disorders.

Participants were children ≥ 7 and < 13 years-old, who underwent polysomnographic evaluation (PSG) at Instituto do Sono (São Paulo) between December 2003 and June 2009. Parents filled in information about weight, height, sleep complaints (The Sleep Disturbance Scale for Children [SDSC])^20^, and sleep patterns (time going to bed and wake up time in weekdays and weekends) on the same night of PSG. The SDSC is the standard procedure in our institution to guide technicians and sleep doctors to analyze the PSG registry. It gives information about 6 domains of sleep (disorders of initiating and maintaining sleep; sleep breathing disorders; disorders of arousal/nightmares; sleep wake transitions disorders; disorders of excessive somnolence; and sleep hyperhidrosis) and it is suitable for a great range of children’s age. Each of these domains have a normal range and the score is not calculated in our institution since the questionnaire’s purpose is to have a better knowledge of the children’s sleep complaints. Information about the specific sleep complaint was taken from the doctor’s referral.

### Inclusion and exclusion criteria

The inclusion criteria were: children whose parents gave permission to use the data for clinical research, undergoing a first evaluation in our institution and have completed the questionnaire regarding sleep hours. Exclusion criteria were: genetic syndromes associated with learning problems, visual or hearing disability, habitual snoring (> 3 times/week), an apnea-hypopnea index ≥ 1, periodic limb movement ≥ 5, elevated chin electromyogram activity (bruxism), and the presence of any epileptiform discharges in an *electroencephalogram*. We also applied the listwise approach for missing data.

### Statistical methods

#### Nutritional status

Parents gave information on weight and height. Nutritional status was defined using body mass index (BMI) for age, or, when the height was not available, by weight for age (W/A). The value available was used as the z-nutritional score. Epi-Info 3.5.3. was used to calculate BMI or W/A z-scores. Z-scores < -2 were classified as malnutrition, ≥ -2 to ≤ +2 normal weight, and ≥ +2 obesity^21^.

#### Income

Income was analyzed as the household income defined by number of minimum wage received by the family. During the period, the mean minimum wage was equivalent to ~U$ 150 / month^22^^,^^23^.

#### Covariates

The following variables were treated as covariates: age, sex, household income (< 2, 2 to 4, 5 to 10 or ≥ 11 times minimum wage), school schedule (does not go to school, attends morning, afternoon or full-time classes), and nutritional z-score. 

Difference of amount of sleep during weekends and during weekdays (weekends - weekdays) was defined as the mean of sleep hours during the weekend minus the mean of the sleep hours during weekdays.

#### Data analysis

We used a listwise approach for missing data. Normality was tested with the Shapiro-Wilk test and since the data was not normally distributed, median and interquartile ranges were used to describe continuous data. Wilcoxon and Mann-Whitney tests were used to compare medians while categorical variables were compared using the qui-squared test (χ^2^).

Sleep duration on weekdays and weekends were calculated for the whole sample and for boys and girls.

Associations between age, sex, household income, school schedule, and nutritional z-score were examined using a generalized linear model (GzLM) with sleep duration on weekdays as the outcome. The same approach was used with the difference between the sleep time during the weekend and weekday.

All statistical tests were two sided and a *p* value < 0.05 was considered statistically significant.

To compare our results with those on the literature, we opt to compare our medians with the means of the other studies giving the assumption that on normal distributions the median, mean, and mode are the same.

#### Ethical

We used questionnaires from families that agreed to share information for future research only. The study protocol was approved by Universidade Bandeirante Anhanguera Ethics Committee (# 855.755 November 03, 2014).

## RESULTS

**Figure 1:**
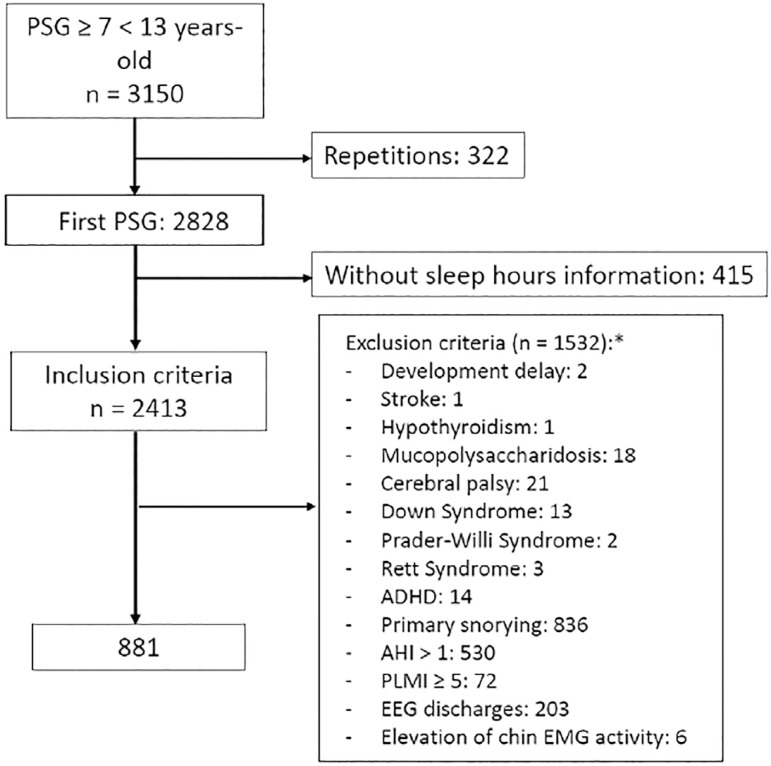
Enrollment flow. PSG = polysomnography, ADHD = Attention Deficit/Hyperactivity Disorder, AHI = apnea-hypopnea index, PLMI = periodic limb movement index, EEG = electroencephalography, EMG = electromyogram, * more than one condition is possible.

After applying the inclusion and exclusion criteria, we included 577 participants. Figure 1 shows the enrollment flow and the doctor’s referral. Missing data were due to non-answer to school schedule (172, [56%]), nutritional status (103, [34%]), and family income (78, [26%]).

Sociodemographic data are shown in Table 1. The patients’ median age was 9.5 years (IQR=3), 61% were boys, and 2.2% obese.

**Table 1:** Socio demographic data

n = 881	Median (P25 - P75)
Age (years)	9.5 (8.1 - 11.1)
Boys (%)	534 (60%)
BMI (n = 537)	18.3 (16 - 21.4)
BMI or W/A z-score (n = 778)	0 (-1.0 - 1.0)
BMI or W/A z-score > + 2	67 (7.6%)
Household income (minimum wage)*	
< 2	360 (41%)
2 to 4	334 (38%)
5 to 10	89 (10%)
≥ 11	20 (2%)
School schedule (%)	
Morning	282 (32%)
Afternoon	264 (30%)
Full-time	120 (14%)
Does not go to school	43 (5%)
Missing	172 (19%)

BMI = body mass index, W/A = weight-for-age.

* Minimum wage = ~U$ 150/ month.

**Figure 2:**
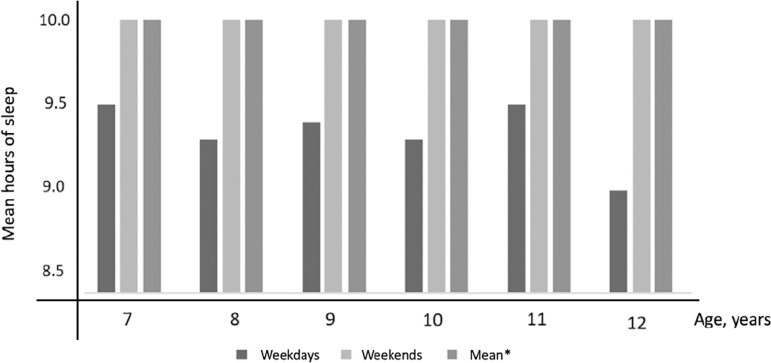
Mean hours of sleep during weekdays, weekends and the mean of (5.weekdays + 2.weekends)/7.

**Figure 3:**
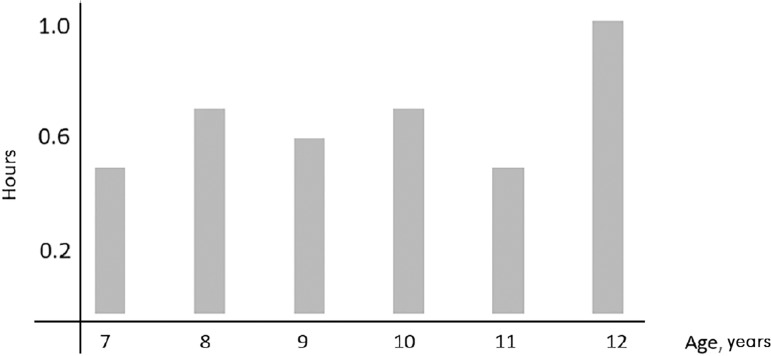
Mean difference in hours from hours of sleep during weekends - weekdays according to age.

Data regarding sleep duration, bedtime, waking up time on both weekdays and weekends, as well as sleep difference weekends - weekdays are shown in Table 2 and Figure 2. Boys and girl’s median bedtime was the same on weekends and weekdays (22:00h), and median wake up time was at 07:00h on weekdays. On weekends, boy’s median woke up time was 08:30h and girls was 09:00h (*p*<0.001). Median sleep duration was longer during weekends compared to weekdays (10h *vs.* 9.5h, *p*<0.001). A difference in sleep weekend - weekdays more than 1.5h was found in 105 girls (46%) and 105 boys (30%), χ^2^=7.17, *p*=0.007. The mean difference in sleep hours during weekends and weekdays divided by age is shown on Figure 3.

**Table 2:** Bedtime, waking up time, sleep duration and sleep debt on weeknights, weekends for all patients and for boys and girls.

	All	Boys	Girls
Bedtime			
Weeknight	22:00 (21:00 - 22:30)*	22:00 (21:00 - 22:07)	22:00 (21:00 - 22:50)
Weekend	22:00 (21:00 - 23:00)	22:00 (21:00 - 23:00)	22:00 (21:00 - 23:00)
Time waking up			
Weeknight	7:00 (6:00 - 9:00)*	7:00 (6:00 - 8:30)	7:00 (6:00 - 9:00)
Weekend	9:00 (8:00 - 10:00)	8:30 (8:00 - 9:30)	9:00 (8:00 - 10:00)*
Sleep duration (hours)			
Weeknight	9.5 (8.5- 10.5)*	9.5 (8.5 - 10.5)	9.5 (8.5 - 10.5)
Weekend	10 (9.5 - 11)	10 (9.5 - 11)	10.5 (10 - 11)*
Sleep debt (hours)	0.5 (0 - 1.5)	0 (0 - 1.5)	1 (0 - 2)*

Sleep debt = median of sleep duration on weekend - median of sleep duration on weeknight.

* *p* < .001 Data shown as Median (P_25_ - P_75_).

The most common sleep complaints were starks or jerks parts of the body before sleeping (19%), grinds teeth (16.5%), and sleep talking^13^. Sleep complaints are described on Table 3.

**Table 3:** Sleep habits and daytime complaints All.

	All	Boys	Girls
Sleeps after 22h on weeknights	264 (30%)	160 (30%)	104 (30%)
Sleeps after 22h on weekend	453 (51.4%)	278 (52.1%)	175 (50.4%)
Sleepiness	77 (8.7%)	36 (6.7%)	41 (11.8%)*
Sleep in inappropriate situations	50 (5.7%)	31 (5.8%)	19 (5.5%)
Sleep at school (≥ 3 times / week)	30 (3.4%)	21 (3.9%)	9 (2.6%)
Learning problems	370 (42%)	248 (46.4%)	122 (35.2%)^†^

* *p* = .01,

† *p* < .01.

In our sample, 46.3% of the children slept less than recommended for their age (9-12 hours of sleep for 6 to 12-year-old children according to The American Academy of Sleep Medicine)^20^. Sleep duration decreases in older children; no sex difference was observed. Bedtime past 22:00h was observed in 171[30%] of children.

For the GzLM, the Gamma distribution was chosen considering the non-normal distribution for sleep hours during the week (Akaike Information Criteria [AIC]_Gamma_=1864, AIC_Linear_=1867, AIC_Tweedie_=1863) and it was the best distribution considering the AIC for the difference in sleep during weekends and weekdays (AIC_Gamma_=799, AIC_Linear_=1904, AIC_Tweedie_=1461). The GzLM showed that age (OR 0.99, _95%_CI 0.99 - 1.00) and school schedule (morning OR 0.90, _95%_CI 0.86 - 0.95, full-time OR 0.94, _95%_CI 0.89 - 0.99, reference: does not go to school) were associated with sleep duration during weekdays. Controlling for age, the effect of it persists (Z=-7.56, *p*<0.001), but there is no interaction of age and period of study (Z=0.12, *p*=0.91). Higher amount of sleep during weekends was associated with age (OR 1.05, _95%_CI 1.01 - 1.10), girls (OR 1.17, _95%_CI 1.02 - 1.34), and school schedule (morning OR 1.67, _95%_CI 1.14 - 2.43, full-time OR 1.81, _95%_CI 1.22 - 2.67). Controlling for age, the effect of it persists (Z=-8.57, *p*<0.001), but there is no interaction of age and period of study (Z=-0.13, *p*=0.90). GzLM for sleep hours during the week is shown on Table 4.

**Table 4:** Generalized Linear Model with sleep hours during the week as outcome. z-BMI = z-score body mass index, MV = minimum wage.

			95% Confidence Interval	
Parameter	B	PR	Lower	Upper
**Constant**	2.4	10.5	9.7	11.3
**Age**	0.0	1.0	1.0	1.0
**Male**	0.0	1.0	1.0	1.0
**z-BMI**	0.0	1.0	1.0	1.0
**Study schedule**				
(ref: do not go to school)				
Afternoon	0.0	1.0	1.0	1.1
Morning	-0.1	0.9	0.9	0.9
Full time	-0.1	0.9	0.9	1.0
**Family income**				
(ref: < 2 MW)				
2 to 4	0.0	1.0	1.0	1.0
5 to 10	0.0	1.0	1.0	1.1
≥ 11	0.0	1.0	1.0	1.1

We found 33 children (6%) that does not go to school. While there are 8 children with 7 years old that might start school next year, we do not have information why these and other children ≥ 8 years old do not go to school.

## DISCUSSION

In our study, according to sleep diary, children went to bed at the same median time on both weekdays and weekends (22:00h). However, the median woke up time was 7:00h on weekdays and 9:00h on weekends. Bedtime on weekdays was later compared to Australia^24^. Wake up time on weekdays was earlier than Spain^25^, later than USA^26^ and a previous Brazilian study^16^. However, in Australia, one study showed a later wake up time^27^ while another one showed a similar wake up time on weekdays for boys^24^. All these studies used sleep questionnaires (Multimedia Activity Recall for Children and Adults, Children’s Sleep Habits Questionnaire, not mentioned, SDSC, and sleep diary, respectively).

Children slept a median of 9.5 hours during weekdays and 10 hours during weekends. Longer sleep duration during weekends has been previously reported^25^^,^^26^. Sleep duration was similar to Australian^24^ and longer than children from USA^26^.

The difference in sleep during weekend - weekdays in our sample (30 minutes) was higher than in Japanese (16 minutes)^28^, Spanish (7 to 19 minutes)^25^ and Australian (16 minutes)^24^ studies but less than a previous Brazilian study with children 10-18 years old (mean sleep difference 0.91±1.67 h in public schools and 1.71±1.41 in private schools)^17^.

The American Academy of Sleep Medicine (AASM) - with the endorsement of the American Academy of Pediatrics, the Sleep Research Society and the American Association of Sleep Technologists - recommends 9-12 hours of sleep for children 6-12 years old^14^. In our sample, almost half of children (46.3%) slept less than the amount recommended for their age.

Most children went to bed before 22h (70%) on weekdays and 50% also follow this pattern on weekends. In our sample, we did not find a sex difference, but a Finnish study with children aged 9.4-13 years showed more boys sleeping after 22h (10% of boys and 5% of girls on weekdays and 66% of boys and 53% of girls on weekends)^29^. A bedtime after 22h has been linked to a higher BMI z-score, overweight and obesity, independent of age, sex, household income, geographical remoteness, and sleep duration in a previous study^30^. Both studies also used sleep questionnaires.

In line with previous studies, sex differences were noted: girls need to compensate more sleep during the weekend (they sleep 30 minutes more on weekends)^18^^,^^25^. Girls also had higher sleepiness complaint (11.8% *vs.* 6.7% of boys, *p*=0.01). A study with adolescents (15 to 20 years old) did not find differences in sleep length in boys and girls, but girls complained more about a higher need of sleep during the week^31^. This subjective sleepiness could explain the higher difference in sleep duration during weekends and weekdays observed in our study. All studies used sleep questionnaires.

As in previous studies, shorter sleep duration was associated with age and morning and full time classes^10^^,^^11^^,^^17^^,^^18^. Higher difference sleep weekends - weekdays was seen in older children, girls, and those attending morning or full time classes^17^^,^^24^^,^^25^^,^^28^. Canet^25^ found that this sleep difference increases in 9-10 year-olds, but decrease in 11-12 year-olds^25^. Woman tend to complain more about their quality of sleep across the lifespan even though they have better objective sleep measurements than men^32^. However, there are data on the literature showing that boys, older age, weekends and low socioeconomic status are associated with shorter time in bed for children^33^.

This study’s strength is the inclusion of children without objective sleep problems (habitual snoring, obstructive sleep apnea, periodic limb movements increase or bruxism register); however, the study has some limitations: it is a cross-sectional study not a cohort study; children were refered to a Sleep Center due to a variety of sleep complaints; parents usually overestimated sleep duration^34^; we do not calculate the questionnaire score in our institution, anthropometric data were reported by parents; we do not have objective home sleep measurements such as actigraphy, and missing data were 14.7%. As we used a questionnaire and not a sleep diary, the bedtime and waketime has to be seen as mean for each child and not the actual time they go to bed or wake up. We use the questionnaire in this paper as it is used in our institution: to guide us knowing the sleep complaints and not as an instrument with clear cutoff points.

Another limitation is the sample analyzed is from ten years ago when new technologies that impact children’s sleep such as on-demand videos, internet videos, and conversations apps were not available. However, this could lead us to a better understanding of the impact of this technologies when we compared this sample to a newer one.

## CONCLUSION

Median bedtime in São Paulo for children 7 to 13 years old was 22h, for woke up 7h on weekdays. On weekends, boys woke up at 08:30h and girls at 9:00h. Shorter sleep duration was associated with age and morning or full-time classes, while higher difference in sleep during weekends - weekdays was associated with older age, girls, and morning or full-time classes. In our study, 46.3% of the children slept less than the 9-12h suggested for 6 to 12-year-old children by the AASM. Sleep schedule interferes with sleep patterns, as children who study in the morning or full time are more prone to sleep less. It is important to give information to parents and teachers about the recommended hours of sleep for children of different ages, and the consequences of lack of sleep, in order to improve the sleep quality of school aged children.

## References

[1] Iglayreger HB, Peterson MD, Liu D, Parker CA, Woolford SJ, Sallinen Gafka BJ (2014). Sleep duration predicts cardiometabolic risk in obese adolescents. J Pediatr..

[2] Michels N, Clays E, De Buyzere M, Vanaelst B, De Henauw S, Sioen I (2013). Children's sleep and autonomic function: low sleep quality has an impact on heart rate variability. Sleep.

[3] Matamura M, Tochigi M, Usami S, Yonehara H, Fukushima M, Nishida A (2014). Associations between sleep habits and mental health status and suicidality in a longitudinal survey of monozygotic twin adolescents. J Sleep Res.

[4] Jang SI, Lee KS, Park EC (2013). Relationship between current sleep duration and past suicidal ideation or attempt among Korean adolescents. J Prev Med Public Health.

[5] Maski KP, Kothare SV (2013). Sleep deprivation and neurobehavioral functioning in children. Int J Psychophysiol.

[6] Narang I, Manlhiot C, Davies-Shaw J, Gibson D, Chahal N, Stearne K (2012). Sleep disturbance and cardiovascular risk in adolescents. CMAJ.

[7] Buchmann A, Ringli M, Kurth S, Schaerer M, Geiger A, Jenni OG (2011). EEG sleep slow-wave activity as a mirror of cortical maturation. Cereb Cortex.

[8] Lustenberger C, Maric A, Dürr R, Achermann P, Huber R (2012). Triangular relationship between sleep spindle activity, general cognitive ability and the efficiency of declarative learning. PLoS One.

[9] Mascetti L, Foret A, Schrouff J, Muto V, Dideberg V, Balteau E (2013). Concurrent synaptic and systems memory consolidation during sleep. J Neurosci.

[10] Iglowstein I, Jenni OG, Molinari L, Largo RH (2003). Sleep duration from infancy to adolescence: reference values and generational trends. Pediatrics.

[11] Liu X, Liu L, Owens JA, Kaplan DL (2005). Sleep patterns and sleep problems among schoolchildren in the United States and China. Pediatrics.

[12] Ohayon MM, Carskadon MA, Guilleminault C, Vitiello MV (2004). Meta-analysis of quantitative sleep parameters from childhood to old age in healthy individuals: developing normative sleep values across the human lifespan. Sleep.

[13] Marinelli M, Sunyer J, Alvarez-Pedrerol M, Iñiguez C, Torrent M, Vioque J (2014). Hours of television viewing and sleep duration in children: a multicenter birth cohort study. JAMA Pediatr.

[14] Paruthi S, Brooks LJ, D'Ambrosio C.Hall WA.Kotagal S.Lloyd RM (2016). Consensus Statement of the American Academy of Sleep Medicine on the Recommended Amount of Sleep for Healthy Children: Methodology and Discussion. J Clin Sleep Med.

[15] Sheldon SH, Ferber R, Kryger MH, Gozal D, eds (2014). Principles and Practice of Peadiatric Sleep Medicine.

[16] Silva TA, Carvalho LB, Silva L, Medeiros M, Natale VB, Carvalho JE (2005). Sleep habits and starting time to school in Brazilian children. Arq Neuropsiquiatr.

[17] (2016). Factors influencing excessive daytime sleepiness in adolescents. J Pediatr (Rio J).

[18] Natal CL, Lourenco TJ, Silva LA, Boscolo RA, Silva A, Tufik S (2009). Gender differences in the sleep habits of 11-13 year olds. Rev Bras Psiquiatr.

[19] Reimão R, de Souza JC, Gaudioso CE, Guerra Hda C, Alves A das C.Oliveira JC (1999). Sleep characteristics in children in the isolated rural African-Brazilian descendant community of Furnas do Dionísio, State of Mato Grosso do Sul, Brazil. Arq Neuropsiquiatr.

[20] Bruni O, Ottaviano S, Guidetti V, Romoli M, Innocenzi M, Cortesi F (1996). The Sleep Disturbance Scale for Children (SDSC). Construction and validation of an instrument to evaluate sleep disturbances in childhood and adolescence. J Sleep Res.

[21] Sociedade Brasileira de Pediatria (SBP), Departamento de Nutrologia (2008). .Obesidade na infância e adolescência: Manual de Orientação.

[22] Guia Trabalhista Tabela dos valores nominais do salário mínimo.

[23] Yahii! Dólar Comercial Oficial - Índice Mensal - 1970 a 2019.

[24] Olds T, Maher C, Blunden S, Matricciani L (2010). Normative data on the sleep habits of Australian children and adolescents. Sleep.

[25] Canet T (2010). Sleep-wake habits in Spanish primary school children. Sleep Med.

[26] National Sleep Foundation (2014). Sleep in America Poll 2014.

[27] Price AM, Brown JE, Bittman M, Wake M, Quach J, Hiscock H (2014). Children's sleep patterns from 0 to 9 years: Australian population longitudinal study. Arch Dis Child.

[28] Takemura T, Funaki K, Kanbayashi T, Kawamoto K, Tsutsui K, Saito Y (2002). Sleep habits of students attending elementary schools, and junior and senior high schools in Akita prefecture. Psychiatry Clin Neurosci.

[29] Saarenpää-Heikkilä OA, Rintahaka PJ, Laippala PJ, Koivikko MJ (1995). Sleep habits and disorders in Finnish schoolchildren. J Sleep Res.

[30] Olds TS, Maher CA, Matricciani L (2011). Sleep duration or bedtime? Exploring the relationship between sleep habits and weight status and activity patterns. Sleep.

[31] Lehto JE, Aho O, Eklund M, Heinaro M, Kettunen S, Peltomäki A (2016). Circadian preferences and sleep in 15- to 20-year old Finnish students. Sleep Sci.

[32] Mong JA, Cusmano DM (2016). Sex differences in sleep: impact of biological sex and sex steroids. Philos Trans R Soc Lond B Biol Sci.

[33] Biggs SN, Lushington K, James Martin A, van den Heuvel C, Declan Kennedy J (2013). Gender, socioeconomic, and ethnic differences in sleep patterns in school-aged children. Sleep Med.

[34] Goodwin JL, Silva GE, Kaemingk KL, Sherrill DL, Morgan WJ, Quan SF (2007). Comparison between reported and recorded total sleep time and sleep latency in 6- to 11-year-old children: the Tucson Children's Assessment of Sleep Apnea Study (TuCASA). Sleep Breath.

